# Use of Automation Technologies and Data Mining in Speech Recognition for Autism

**DOI:** 10.1002/brb3.71229

**Published:** 2026-01-28

**Authors:** Rongjie Mao, Yuncheng Zhu

**Affiliations:** ^1^ Department of Child and Adolescent Psychiatry Shanghai Hongkou Mental Health Center Shanghai China; ^2^ Mental Health Center Affiliated to School of Medicine Shanghai University Shanghai China; ^3^ Clinical Research Center For Mental Health, Mental Health Center Affiliated to School of Medicine (Shanghai Hongkou Mental Health Center) Shanghai University Shanghai China

**Keywords:** autism spectrum disorder, automation technologies, data mining, speech recognition

## Abstract

**Introduction:**

Early identification of autism spectrum disorder (ASD) is critical for improving long‐term outcomes, and speech offers a noninvasive source of clinically relevant biomarkers. However, manual speech analysis is time‐consuming and difficult to scale. With advances in digital recording, signal processing, and artificial intelligence, researchers have increasingly deployed automated tools and data‐mining methods to characterize speech and language in ASD.

**Methods:**

This structured narrative review summarizes methodological developments in speech‐based ASD assessment from 1994 to 2025, spanning diverse tasks and recording settings and focusing on automated tools, data‐mining methods, and their clinical translation. We first consider core automated toolchains, including LENA, Praat, HTK/FAVE, CMU Sphinx, Kaldi, AutoSALT, openSMILE/eGeMAPS, diarization systems, and foundation‐model ASR systems (e.g., Whisper), as well as modern self‐supervised encoders such as wav2vec 2.0 and TRILLsson. Their typical use cases, psychometric properties, and limitations are highlighted. We then chart the progression of data‐mining and machine‐learning approaches from early logistic regression and clustering, through regularized regression, SVMs, and tree ensembles, to CNN/LSTM sequence models and transformer‐based text and speech models (e.g., BERT, LLMs).

**Results:**

Across these stages, automated indices of prosody, voice quality, linguistic content, and interactional behavior show moderate‐to‐high accuracy for ASD detection and meaningful associations with clinician‐rated severity. Nonetheless, various problems persist: performance often degrades across languages, ages, tasks, and recording settings; evaluation and reporting remain heterogeneous; datasets are typically small and single‐site; and privacy, fairness, interpretability, and computational efficiency pose persistent barriers to deployment, highlighting the need for target‐context benchmarking and pre‐specified evaluation/reporting.

**Conclusion:**

We outline three priority strategies to guide future work toward scalable, clinically credible ASD speech assessment and longitudinal monitoring: optimize and integrate existing toolchains, enable global yet privacy‐preserving data sharing, and leverage cross‐domain innovations in enhancement, label efficiency, and explainable, edge‐ready AI.

## Graphical abstract

Pipeline analyzes clinical and naturalistic speech using LENA, wav2vec 2.0, and foundation‐model ASR (Whisper) to enable scalable ASD detection and severity estimation. Future work integrates benchmarking, privacy‐preserving collaboration (federated learning), and explainable, edge‐ready AI for clinically credible assessment and longitudinal monitoring.

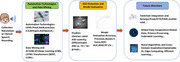

**Abbreviations**
ACPU − C V: average count per utterance‐consonants + vowels
ADOS: autism diagnostic observation schedule
ALBERT: a lite bidirectional encoder representations from transformers
ALM: automated language measure
ASD: autism spectrum disorder
ASR: automatic speech recognition
AUC: area under the receiver‐operating‐characteristic curve
AVA‐DA: automated vocalization analysis‐developmental age
AVM: automated voice measure
AWC: adult word count
BERT: bidirectional encoder representations from transformers
BiLSTM: bidirectional long short‐term memory network
CA: cluster analysis
CCC: concordance correlation coefficient
CNN: convolutional neural network
CPM: communication units per minute
CPP: cepstral peak prominence
CTC: conversational turn count
CV: cross‐validation
CVC: child vocalization count
DNN: deep neural network
DT: decision tree
eGeMAPS: extended Geneva Minimalistic Acoustic Parameter Set
ELMo: embeddings from language models
F0: fundamental frequency
FAVE: forced alignment and vowel extraction
GAMI‐Net: generalized additive models with structured interactions network; an explainable neural network based on generalized additive models with structured interactions
GAN: generative adversarial network
GPT: generative pre‐trained transformer
HC: hierarchical clustering
HMM: hidden Markov model
HNR: harmonic‐to‐noise ratio
HTK: Hidden Markov Model Toolkit
ICC: intraclass correlation coefficient
IMFs: intrinsic mode functions
IVD: infraphonological vocal development
i‐vectors: low‐dimensional utterance‐level representations derived from factor analysis, widely used in speaker recognition
KLUE: Korean Language Understanding Evaluation
LDA: linear discriminant analysis
LENA: language environment analysis system
LIME: local interpretable model‐agnostic explanations
LLM: large language model
LOOCV: leave‐one‐out cross‐validation
LR: logistic regression
LSTM: long short‐term memory
MAE: mean absolute error
MFCC: Mel‐frequency cepstral coefficients
MLR: multiple linear regression
MLUM: mean length of utterance in morphemes
NDWR: number of distinct word roots
NLP: natural language processing
NWR: non‐word repetition
PCA: principal component analysis
PER: phoneme error rate
Praat: open‐source software package widely used for phonetic and speech analysis
R^2^
: coefficient of determination
RF: random forest
Ridge: ridge regression
RoBERTa: a robustly optimized bert pretraining approach
RRB: restricted and repetitive behaviors
SALT: systematic analysis of language transcripts
SCQ: social communication questionnaire
SED: speech activity/segmentation features
SIT: simulated interaction task
SLI/ALI/ALN: specific language impairment/autism + language impairment / autism without language impairment
SNR: signal‐to‐noise ratio
Sphinx: open‐source speech‐recognition toolkit designed for automatic transcription and acoustic modeling
SSL: self‐supervised learning
SVM: support vector machine
SVR: support vector regression
TD: typically developing
TinyML: tiny machine learning (resource‐constrained on‐device machine learning)
TRILLsson: distilled convolutional neural network speech embeddings
TTS: text‐to‐speech
USE: universal sentence encoder
wav2vec 2.0: self‐supervised model for speech representation learning from raw audio
WER: word error rate
Whisper: a large‐scale multilingual encoder‐decoder ASR model (OpenAI)
XAI: explainable artificial intelligence
XGBoost: extreme gradient boosting (gradient‐boosted decision tree algorithm)

## 1 Introduction

Autism spectrum disorder (ASD) is a neurodevelopmental condition characterized by deficits in social communication deficits and the presence of repetitive, stereotyped behaviors (Hirota and King 2023). Early identification and intervention are critical for improving long‐term developmental outcomes (Lord et al. 2020). Speech analysis has emerged as a key method for identifying individuals with ASD (Rogers et al. 2024), focusing mainly on three dimensions (Eni et al. 2025; Vogindroukas et al. 2022): (1) prosodic and voice quality features, such as intonation, speech rate, and formant frequencies, reflecting emotional expression and the physical properties of the voice; (2) linguistic content features, including lexical diversity and grammatical structure, assessing the level of language development; and (3) interactional features, such as turn‐taking and vocal responsiveness, revealing functional characteristics in socially interactive speech.

**FIGURE 1:**
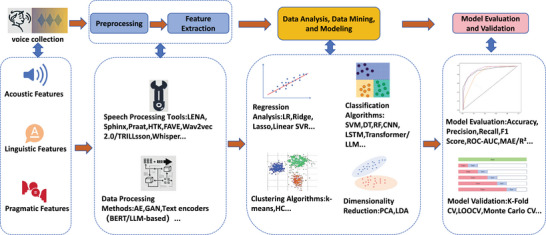
A comprehensive computational pipeline for automated speech recognition and analysis in ASD. Abbreviations not defined in the main text are explained below; all others are defined at first mention in the article: AE, auto‐encoder; LR, Logistic Regression; Lasso, Lasso Regression; Ridge, Ridge Regression; DT, Decision trees; RF, Random forests; k‐means, k‐means clustering; HC, Hierarchical Clustering; CV, Cross‐validation. Abstract: This flowchart illustrates the end‐to‐end process of leveraging automation technologies and data mining to extract clinically relevant biomarkers from speech for ASD detection and assessment.

Our review conceptualizes ASD speech recognition as an integrated pipeline (Figure 1).

Test: The pipeline begins with (1) Voice Collection of speech samples from individuals with ASD. The raw audio then undergoes (2) Preprocessing to be prepared for analysis. Subsequently, a suite of (3) Automated Speech Processing Tools (e.g., LENA, Praat, Wav2vec 2.0) is employed for (4) Feature Extraction, deriving acoustic, linguistic, and pragmatic features. These features fuel the core (5) Data Analysis, Data Mining, and Modeling stage, which encompasses a wide array of methods‐from regression and clustering to deep learning and Transformers‐to build predictive models. The pipeline concludes with (6) Model Evaluation and Validation, ensuring robustness and clinical relevance through rigorous performance metrics and validation techniques.

We conducted a structured narrative review focused on methodological advances in publications from January 1994 to October 2025. Our search included sources indexed in PubMed, Web of Science, and IEEE Xplore complemented by cross‐referencing of seminal studies and recent conference proceedings. The search combined autism‐related keywords (e.g., “autism,” “autism spectrum disorder,” “ASD”) with speech and computational‐analytics terms (e.g., “speech,” “voice,” “prosody,” “acoustic,” “automatic speech recognition,” “ASR[automatic speech recognition],” “speaker diarization,” “machine learning,” “deep learning,” “neural network,” “self‐supervised,” “wav2vec,” “bidirectional encoder representations from transformers [BERT],” “transformer”) with Boolean strings adapted to each database. We prioritized peer‐reviewed articles reporting quantitative performance or reliability for speech‐based ASD assessment tasks and excluded works on non‐speech biomarkers, duplicate reports, and studies without evaluable metrics. Given the heterogeneity of tasks and outcomes, we did not perform meta‐analysis; instead, we synthesized trends, methodological inflection points, and gaps relevant to clinical translation.

Having outlined our search strategy and inclusion criteria, we next summarize the main classes of automated speech analysis tools that underpin ASD speech analytics, before turning to data‐mining and machine‐learning methods.

## 2 Automated Speech Analysis Tools

### 2.1 LENA (Language Environment Analysis)

Introduced around 2004, LENA is among the most widely used tools for studying early speech and language environments of children with autism (VanDam and Yoshinaga‐Itano 2019). It records a child's daily acoustic environment in the home or school and automatically identifies speakers to estimate adult word count, child vocalizations, and conversational turns. LENA recordings can be further processed to derive additional acoustic parameters such as rhythm, syllabicity, spectral tilt, and pitch‐control features, providing a richer view of vocal development (Oller et al. 2010). In an early study, LENA achieved 85%–90% accuracy in detecting natural speech in children with ASD (Xu et al. 2009). LENA metrics have also shown good test‐retest reliability and correlations with later language outcomes in toddlers (Woynaroski et al. 2017; Yoder et al. 2013). Recent psychometric research has established the reliability and clinical convergent validity of the full LENA variable set in autistic toddlers, supporting its utility as a naturalistic language assessment tool and laying foundations for longitudinal applications (Nadwodny et al. 2025).

Despite these strengths, LENA's accuracy tends to decrease in multi‐speaker or noisy settings and may drop below 50% for children aged five years or older, with misclassification rates increasing significantly with age (Jones et al. 2019). Importantly, the same study further showed that LENA's automated estimates are highly sensitive to recording conditions (e.g., device placement), underscoring the need to clearly report recording procedures and to validate performance in the target setting. Human‐coded speech‐quality measures can also capture communicative nuances that current automated algorithms such as LENA may miss (McDaniel et al. 2020). Putnam et al.'s (2025) comprehensive systematic review summarized LENA's major advantages‐ecological validity, cost‐effectiveness, and clinical application potential‐and its persistent limitations: reduced speaker‐identification accuracy, variability in recording conditions, and limited generalizability across populations and developmental stages. In practice, LENA is a foundational recording and diarization platform, complemented by finer‐grained acoustic analyses (e.g., Praat) to characterize prosody and voice quality.

### 2.2 Praat, HTK, FAVE, and Sphinx

Since the early 2010s, autism speech research has adopted a set of widely used, complementary speech analysis tools. For acoustic analysis, autism researchers commonly use Praat to extract prosodic and voice‐quality features, including fundamental frequency (F0), speech rate, rhythm, intensity, jitter, shimmer, harmonic‐to‐noise ratio (HNR), and formant‐based measures. In representative interview data from the Autism Diagnostic Observation Schedule (ADOS), these acoustic markers have been found to correlate with clinician‐rated symptom severity (e.g., flatter turn‐end pitch slope [*r* = −0.68], higher jitter [*r* = 0.38], and reduced HNR [*r* = −0.38]) (Bone et al. 2014; see also Patel et al. 2020; Wynn et al. 2022). However, Praat requires manual parameter calibration and quality control, and its outputs are sensitive to background noise, overlapping speech, and the atypical phonation typical of autistic speakers (Bone et al. 2014; Briend et al. 2023; Esposito et al. 2013). Despite these limitations, it remains a reliable, transparent tool for fine‐grained acoustic phenotyping and is frequently paired with automated pipelines for cross‐validation and interpretability.

Hidden Markov Model Toolkit (HTL) and forced alignment and vowel extraction (FAVE) are primarily alignment toolkits providing time‐accurate boundaries for downstream acoustic/linguistic analyses. HTK aligns audio to transcripts, enabling time‐locked segmental and suprasegmental measures. In specific tasks/samples, such alignments have supported links between cues such as turn‐end pitch slope or rhythm and ASD symptom severity (Bone et al. 2014; Patel et al. 2020). Limitations of HTK include acoustic models tuned to adult American English, reduced accuracy for child/atypical voices and for noisy or overlapping speech, and dependence on transcript/diarization quality—thus, manual quality control is often needed. FAVE adds automatic vowel (F1/F2) measurement, facilitating large‐scale analyses of vowel space, rhythm, and articulatory precision in English corpora. However, it typically requires adaptation for non‐English or clinical populations. Despite these constraints, both tools remain backbone components for time‐precise, reproducible ASD speech pipelines.

As alignment toolchains became established, researchers also adopted automatic speech recognition (ASR) frameworks such as CMU Sphinx to support fully automated processing of naturalistic recordings. In ASD cohorts, Sphinx has been used‐often alongside LENA‐to derive automatic transcripts and speech‐quantity/timing indices (e.g., child vocalizations, conversational turns) and to support predictive modeling. Infraphonological vocal development (IVD) and average count per utterance − consonants + vowels (ACPU ‐ C + V) scores predicted later expressive vocabulary in preverbal toddlers (r = 0.51 and r = 0.55, respectively; Woynaroski et al. 2017), although human‐coded speech‐quality measures were subsequently found to be moderately stronger predictors in the same age range (pseudo‐*R* ^2^ ≥ 0.25 vs. 0.19/0.08; McDaniel et al. 2020). Stability can vary by variable and cohort (e.g., sibling designs), underscoring the need for careful quality control and repeated measurements (Markfeld et al. 2023; see Table S1). Accuracy declines in noisy or multi‐speaker settings and with atypical phonation, reflecting training on adult English speech and dependencies on diarization/transcript quality. Sphinx nonetheless remains a practical foundation for large‐scale automatic analysis and a bridge toward newer, more robust deep‐learning ASR pipelines.

### 2.3 Modern Speech Analysis Frameworks (wav2vec 2.0, TRILLsson, Whisper, and ASDSpeech)

Since the early 2020s, ASD speech research has increasingly adopted data‐driven methods that reduce reliance on manually designed features. A key trend is the use of pretrained acoustic encoders, including self‐supervised models such as wav2vec 2.0 and lightweight distilled models such as TRILLsson, which provide general‐purpose speech representations that can be fine‐tuned with relatively few clinical labels (Shor and Venugopalan 2022). In family and semi‐structured child speech, wav2vec 2.0 achieved 76.9% accuracy, compared with 79.3% for a CNN and 69.7% for a random forest, suggesting that self‐supervised representations are label‐efficient but still require adaptation for noisy, atypical child phonation (Chi et al. 2022; Cai et al. 2024). The Noor Project (Al Futaisi et al. 2025) reported effective ASD recognition with as little as ∼30 min of labeled child speech, highlighting the value of transfer learning for low‐resource clinical settings. However, such end‐to‐end pipelines often function as “black boxes,” with limited transparency and clinical interpretability.

In parallel, modern pipelines increasingly incorporate foundation‐model ASR to derive transcripts for downstream linguistic and conversational analyses. Whisper is a widely used end‐to‐end ASR model, but its accuracy can degrade on disfluent or atypical child speech and may under‐represent clinically salient phenomena such as pauses, repetitions, and fillers (Radford et al. 2023). In classroom conversations with children with verbal ASD, for example, Whisper achieved a low word error rate (WER) for mild autism (median ≈ 5%) but a higher and more variable WER for moderate autism (median ≈ 23%), underscoring the need for target‐context error profiling and for propagating ASR uncertainty into downstream analyses (Muna and Setiawan 2025).

Clinically oriented supervised systems emphasize interpretability and alignment with diagnostic tools. ASDSpeech (Eni et al. 2025) is an open‐source model trained on 99,193 annotated vocalizations from 197 children across 258 ADOS‐2 assessments. Using handcrafted acoustic and conversational features (e.g., pitch variability, vocalization rate), it correlates moderately with clinician‐rated ADOS‐2 Social Affect scores (r ≈ 0.50–0.60, *p* < 0.0001), but shows weak performance on Restricted and Repetitive Behaviors (RRB), underscoring that speech alone cannot capture the full ASD phenotype (Eni et al. 2025).

Seeking a tool for end‐to‐end estimation of clinical severity without manual transcripts, researchers are exploring emerging cascaded and multimodal pipelines that fuse raw‐audio SSL embeddings with ASR‐derived text and conversational cues. As the performance of several prototypes awaits peer review, we note only that this direction shows promise for future validation and multilingual, multi‐site testing (Mun et al. 2025). In parallel, privacy‐preserving and explainable multimodal frameworks (e.g., GAMI‐Net) aim to integrate speech with other behavioral signals to deliver transparent, clinically useful decision support; they also require multi‐site, multilingual validation (Malik et al. 2025).

### 2.4 Other Widely Used Toolkits

Kaldi is an open‐source ASR toolkit providing frame‐aligned transcripts and error metrics (WER/phoneme error rate [PER]). In ASD research, Kaldi enables automated scoring of nonword/sentence repetition and objective phoneme‐level analyses. Using Kaldi (Gaussian Mixture Model/Hidden Markov Model [GMM/HMM] and deep neural network [DNN] acoustic models)with transfer learning and augmentation, studies report strong agreement with clinician scores (e.g., *r* ≈ 0.85; mean absolute error [MAD] ≈ 0.04) and reduced WER (≈ 29.4% → 26.2%), although irrelevant segments still require manual removal and performance can degrade with atypical child phonation (Asgari et al. 2017; Gale et al. 2019).

Systematic Analysis of Language Transcripts (SALT) is a standardized language‐sample analysis framework. In the form of AutoSALT, it can be used to generate automated language measures (ALMs) such as mean length of utterance in morphemes (MLUM), number of distinct word roots (NDWR), and c‐units per minute. In ASD cohorts, AutoSALT has shown good test‐retest reliability (concordance correlation coefficient [CCC] ≈ 0.73–0.88) but ALM performance varies across tasks (e.g., interview, conversation, narration), underscoring the need to report context and combine ALMs with acoustic features for robust classification (MacFarlane et al. 2023; MacFarlane et al. 2022).

OpenSMILE is a widely used feature‐extraction toolkit, while eGeMAPS (extended Geneva Minimalistic Acoustic Parameter Set) is a compact paralinguistic set for measuring F0, jitter/shimmer, HNR, MFCC (mel‐frequency cepstral coefficients), and energy. In ASD speech research, eGeMAPS provides a reliable baseline: coupled with deep models, it achieves moderate discrimination in infants, while with classical support vector machine (SVM) and decision tree classifiers, it reveals atypical prosody/voice quality in school‐aged children. Sensitivity to noise and overlap remains a limitation, necessitating careful quality control and, where possible, fusion with higher‐level features (Beccaria et al. 2022; Lee et al. 2020; Mencattini et al. 2018).

DiarTK provides speaker diarization for multi‐speaker clinical recordings (e.g., ADOS), enabling turn segmentation/labeling and extraction of turn‐taking, initiation, and duration features. When combined with CNN or Long Short‐Term Memory (LSTM) models, diarized features have predicted ADOS‐2 social affect with moderate accuracy (*R* ^2^ [coefficient of determination] ≈ 0.40), though manual checks and small cohorts remain common (Sadiq et al. 2019).

Supplementary Table S1 consolidates the main automated speech analysis tools used in ASD research, summarizing their speech contexts, target populations, key findings, strengths, limitations, and best‐fit use cases.

## 3 Data Mining and Machine Learning Methods

This section outlines the historical development of data‐mining and machine‐learning approaches in ASD speech analytics, highlighting key methodological advances, representative applications, and their respective strengths and limitations across different stages of technological progress.

### 3.1 1990s: Early Data Mining via Manual Pipelines

During the 1990s, when automated speech‐analysis tools were largely unavailable, ASD language and speech research began to adopt fundamental concepts from the field of Knowledge Discovery in Databases (Fayyad et al. 1996). Researchers applied relatively simple yet scalable statistical techniques such as logistic regression and cluster analysis to manually curate behavioral and linguistic measures (Eaves et al. 1994; Eisenmajer et al. 1996). These early efforts facilitated preliminary pattern discovery and quantitative exploration of speech‐related features associated with autism. Their main advantages included straightforward implementation, computational efficiency, and interpretability. However, inherent limitations included reliance on small, manually annotated datasets and handcrafted features, and inadequate capacity to model nonlinear, high‐dimensional, or temporally dynamic characteristics of natural speech.

### 3.2 2000s–2010s: Regularized Linear Models, Kernel/Ensemble Learners, and Shallow Temporal Models

As larger datasets such as LENA naturalistic recordings and structured tasks like ADOS became available, ASD speech analytics entered a new phase by adopting classical machine‐learning methods. These approaches included regularized regression (lasso/ridge regression) to reduce overfitting in small, high‐dimensional datasets (Asgari et al. 2017), SVMs with nonlinear kernels to capture complex feature relationships (Prud'hommeaux and Rouhizadeh 2012), and tree‐based ensembles (e.g., random forests) to stabilize decision‐tree variability (Drimalla et al. 2020). Dimensionality‐reduction techniques such as principal component analysis and linear discriminant analysis were applied to manage correlated acoustic measures (Oller et al. 2010), while hidden Markov models (HMMs) provided preliminary modeling of temporal patterns (Prud'hommeaux and Rouhizadeh 2012). Cross‐validation strategies such as LOOCV (Leave‐One‐Out Cross‐Validation) and k‐fold became routine, improving the reliability of reported results and marking a transition toward more systematic evaluation protocols (Beccaria et al. 2022; Manigault et al. 2023).

Representative studies during this period established foundations for automated acoustic and linguistic assessment. Oller et al. (2010) used LENA's fully automated acoustic classifier based on 12 vocal‐development parameters (e.g., rhythm, pitch control, spectral tilt) to distinguish ASD from typically developing (TD) children, achieving sensitivity = 0.75 and specificity = 0.98. Prud'hommeaux and Rouhizadeh (2012) employed word alignment implemented with HMMs plus log‐odds lexical features and an SVM classifier to quantify pragmatic atypicality: moderate discrimination was obtained in narrative tasks (area under the curve [AUC] ≈ 0.72). Asgari et al. (2017) applied ridge, lasso, and support vector regressions to automatically score nonword‐repetition accuracy, reporting a high correlation (*r* = 0.85) with clinician ratings. Cross‐linguistic research by Lau et al. (2022) further showed that rhythm‐based temporal features were more stable than intonation‐based cues across languages (English and Cantonese), highlighting that cross‐language variability inhibits generalization. Overall, these studies show encouraging performance but remain constrained by small, demographically narrow samples and limited training data, with generalizability issues discussed in detail in Section 4.1.

Meanwhile, pragmatic mid‐spectrum approaches emerged between handcrafted pipelines and fully end‐to‐end models. Time‐varying harmonic models paired with SVMs captured prosodic/voice‐quality dynamics in ADOS conversations (AUC ≈ 0.82–0.83), albeit with task sensitivity (Asgari et al. 2021). As an unsupervised pre‐step, k‐means clustering supported triage and class discovery; in a 6.3‐12‐year‐old cohort completing non‐word/sentence repetition tasks, it reached 91% accuracy (89% sensitivity; 94% specificity; Monte‐Carlo CV), facilitating stratification before supervised learning (Briend et al. 2023). These mid‐spectrum strategies foreshadowed the subsequent turn to pretrained acoustic encoders and Transformer‐based text models (see Section 3.4).

Overall, this period yielded improved control of model bias and variance, enhanced interpretability, and the emergence of reproducible analytic pipelines integrating alignment, feature extraction, and validation. Yet several limitations persisted: models still relied heavily on handcrafted features and manual annotations, struggled to capture long‐range temporal dynamics, and performed inconsistently across datasets owing to small sample sizes and language‐ or task‐specific variability (Asgari et al. 2017; Lau et al. 2022; MacFarlane et al. 2022; Oller et al. 2010; Prud'hommeaux and Rouhizadeh 2012). These constraints motivated the subsequent transition toward deep, self‐supervised learning paradigms capable of leveraging larger, more heterogeneous speech corpora for robust ASD modeling.

### 3.3 Late 2010s–Early 2020s: CNN/LSTM Sequence Models for Dynamic Speech Features

By the late 2010s, deep‐learning architectures like CNN and LSTM models became increasingly common in ASD speech analytics, enabling better modeling of dynamic prosodic patterns and articulatory dynamics compared to previous feature‐based methods (Kato et al. 2022). Sadiq et al. (2019) introduced one of the first large‐scale deep pipelines, combining CNN and LSTM layers on diarized ADOS interviews to predict ADOS‐2 Social Affect scores (*R* ^2^ = 0.40). Although this approach improved predictive accuracy compared with static acoustic features, it remained constrained by limited sample size, manual labeling requirements, and potential overfitting. Similarly, Lee et al. (2020) used autoencoder and Bi‐LSTM models on openSMILE‐derived features for ASD versus TD classification: although moderate accuracy was achieved (≈ 68%), challenges included data imbalance, background noise, and restricted interpretability—all common limitations of early deep‐learning applications in small, heterogeneous datasets. Further extending this acoustic focus, Wynn et al. (2022) used deep networks on Praat‐derived formant trajectories, revealing consistent deficits in articulatory precision among autistic children and adults. This evidenced deep models’ sensitivity to subtle articulatory cues, despite their continued dependence on extensive preprocessing and high‐quality recordings.

In parallel, textual and multimodal approaches also advanced. Kato et al. (2022) used bidirectional long short‐term memory (BiLSTM) and DNN models on lexicogrammatical features, achieving strong classification (F1 = 0.88) and reducing the need for manual annotation; however, the model's generalizability was limited by reliance on linguistically homogeneous data. In multimodal work, MacFarlane et al. (2022) integrated automated language measures (ALMs) and automated voice measures (AVMs) in a hybrid DNN‐SVM pipeline, achieving high accuracy (≈ 87%, AUC = 0.92) and demonstrating that feature fusion outperforms unimodal methods. However, the dataset was biased toward English speakers with normal IQ, and model interpretability was limited. Additionally, ASR transfer learning using Kaldi (DNN/GMM‐HMM) proved effective with limited data, lowering WER from 29.38% to 26.21% on child non‐word repetition tasks and revealing diagnosis‐specific error patterns (Gale et al. 2019).

Overall, the integration of CNN and LSTM architectures represented a major methodological advancement in ASD speech analytics by enabling end‐to‐end learning of sequential acoustic and linguistic features and reducing reliance on handcrafted measures. Nonetheless, these models were typically data‐hungry and domain‐specific, relying on large amounts of carefully annotated speech and often offering limited interpretability and reproducibility in the absence of standardized evaluation benchmarks. (Kato et al. 2022; Lee et al. 2020; MacFarlane et al. 2022; Sadiq et al. 2019; Wynn et al. 2022).

### 3.4 Transformer‐Based Models (BERT, XLNet, and GPT)

Before transformer‐based models became standard, studies utilized deep text embeddings like embeddings from language models (ELMo) and the universal sentence encoder (USE) with shallow classifiers (e.g., SVM, XGBoost [extreme gradient boosting]), yielding modest improvements over traditional bag‐of‐words and handcrafted features (e.g., Wawer and Chojnicka 2022). These “pre‐transformer” pipelines laid foundations for BERT‐style models and large language models (LLMs), which capture richer semantic and pragmatic information directly from text. Transformer architectures replaced recurrence with multi‐head self‐attention, enabling parallel training and long‐range contextual modeling (Papanastasiou et al. 2024). Key developments include BERT, which introduced bidirectional masked‐language pretraining (Devlin et al. 2019); XLNet, which generalized autoregressive pretraining via permutation language modeling to capture bidirectional context without masking (Yang et al. 2019); and GPT‐3/4 (generative pre‐trained transformer), which scaled autoregressive transformers for few‐/zero‐shot task transfer (Brown et al. 2020; Achiam et al. 2023). These capabilities are particularly valuable for ASD research, as many socially relevant markers (e.g., atypical discourse, echoic repetition, and pronoun use) are semantic‐pragmatic in nature and thus more easily captured by long‐range contextual language representations.

Compared with earlier feature‐engineered or shallow‐sequence models, Transformer encoders capture discourse‐level dependencies, long‐distance co‐reference, and lexical‐semantic nuance, enabling detection of pragmatic anomalies salient to ASD (Devlin et al. 2019; Yang et al. 2019). Studies using Transformer embeddings on clinical or narrative transcripts demonstrate gains over traditional text features. In book‐narrative retellings, deep text embeddings outperformed human raters yet remained below gold‐standard instruments (Wawer and Chojnicka 2022); applied to a larger pediatric dataset, BERT embeddings with gradient‐boosting achieved around 96% accuracy in distinguishing ASD from TD, underscoring the value of deep semantics for detecting developmental language patterns (Themistocleous et al. 2024). Extending beyond controlled narratives to more interactive speech, recent LLM‐based studies indicate that Transformer models can surface hallmark language phenomena and improve classification in real‐world dialogue. For instance, ChatGPT (a conversational generative pre‐trained transformer [GPT] model) detected echolalia, stereotyped media quoting, and atypical phrasing in interactive adult speech, and surpassed each of the standalone baselines (BERT, XLNet, a lite bidirectional encoder representations from transformers [ALBERT]) by 12–22 percentage points in accuracy and F1 score (the harmonic mean of precision and recall), albeit in a demographically narrow sample (Hu et al. 2024). Collectively, the evidence supports the effectiveness of Transformers for semantic‐pragmatic profiling of ASD while highlighting dependence on transcript quality and cohort scope (Hu et al. 2024; Themistocleous et al. 2024; Wawer and Chojnicka 2022).

However, current studies remain highly sensitive to transcript quality: performance is strongest when models are trained and tested on carefully curated manual transcripts and often degrades when relying on noisier ASR output, especially for child or atypical speech. In addition, many systems offer limited interpretability and demand substantial computational resources, raising concerns about transparency, deployment feasibility, and equity. Moreover, well‐documented risks of general‐purpose LLMs—such as bias, hallucination, and privacy leakage from training data and user prompts—carry over to ASD applications and therefore require explicit safeguards (Gadotti et al. 2024; Keykha et al. 2024).

Supplementary Table S2 summarizes data mining and machine learning methods in ASD research, comparing their speech contexts, target populations, key findings, strengths, limitations, and optimal use cases.

## 4 Recurrent Challenges

### 4.1 Generalizability Across Language, Age, Task, and Setting

Performance often attenuates when models are applied to different languages, demographics, and acoustic contexts. Cross‐linguistic meta‐ and comparative studies suggest that rhythm‐based temporal cues are more stable than intonation‐only markers, but language‐specific prosody and demographic imbalance still limit robustness (Fusaroli et al. 2022; Lau et al. 2022). Task heterogeneity further undermines stability: ALMs can be reliable within a task but not across ADOS interviews, conversation, and narration (MacFarlane et al. 2023). Importantly, such context effects can be substantial even within a single “standardized” assessment. For example, within the ADOS, some activities are explicitly language‐demanding for verbal children (e.g., conversational prompts or narrative tasks), whereas others may elicit minimal speech (e.g., bubbles/balloons). Analyses restricted to selective activities or time windows can therefore yield markedly different automated measures and model performance (Lord et al. 2012; MacFarlane et al. 2023). We recommend explicitly documenting the ADOS module/activities (or analogous segments), segment inclusion/exclusion rules, and key recording conditions to support interpretability and cross‐study comparison. Reliability is also sample‐dependent: some automated vocal indices stabilize within a single day for toddlers (Yoder et al. 2013), whereas multiple recordings are needed for other populations (Markfeld et al. 2023). Age effects are nontrivial: systems tuned to toddlers (e.g., 16–48 months) perform well (Oller et al. 2010), but detection accuracy drops in children ≥ 5 years (Jones et al. 2019). Moreover, human‐coded quality measures can still surpass automated ones for predicting language outcomes (McDaniel et al. 2020). Finally, household noise and multi‐speaker overlap degrade forced alignment and ASR accuracy across common toolchains, highlighting sensitivity to recording conditions (Bone et al. 2014; Jones et al. 2019; Rybner et al. 2022).

### 4.2 Evaluation and Reporting Comparability

Studies report heterogeneous metrics‐accuracy, AUC, sensitivity/specificity, *R* ^2^, MAE, and WER/PER—on small, single‐site samples with differing ages, languages, and tasks, which hinders meta‐analytic synthesis (Asgari et al. 2017; MacFarlane et al. 2022; MacFarlane et al. 2023; Oller et al. 2010). Few works use prespecified external test sets, cross‐site splits, or calibrated symptom severity scores; uncertainty intervals are inconsistently reported; and benchmark tasks remain fragmented across corpora (Putnam et al. 2025; Rybner et al. 2022). Without harmonized outcome definitions and validation protocols, it is difficult to compare models or determine whether improvements reflect better modeling or dataset idiosyncrasies. More broadly, given the high‐dimensional feature spaces and many analytic degrees of freedom in ASD speech analytics, the field is particularly vulnerable to analytic flexibility and selective reporting. We therefore encourage a clearer separation between exploratory analyses (hypothesis generation, feature/model search) and confirmatory evaluation using pre‐specified primary endpoints, locked preprocessing/modeling pipelines, and independent replication (e.g., held‐out sites or newly collected cohorts). Where feasible, preregistration and transparent reporting of all tried analyses can further improve reproducibility.

### 4.3 Multimodal Integration and Clinically Anchored Endpoints

Multimodal fusion—combining ALM with AVM—typically outperforms unimodal variants, but gains depend on transcript quality, task standardization, and sample composition (MacFarlane et al. 2022). Deep sequence models can map audio to clinician‐rated constructs (e.g., ADOS‐2 social affect), yet training datasets are small, labels are costly, and external validation is sparse (Sadiq et al. 2019). Self‐supervised encoders can now estimate symptom severity from raw audio at scale, but calibration, uncertainty reporting, and cross‐site harmonization remain uncommon (Chi et al. 2022; Eni et al. 2025; Lee et al. 2020). In short, interpretability and transportability lag behind headline performance, leaving a gap between research prototypes and deployable clinical endpoints.

### 4.4 Data Resources, Documentation, and Infrastructure

High‐quality, large‐scale, clinically annotated datasets remain scarce, especially those spanning multiple ages, languages, tasks, and recording settings. Available corpora are often small, single‐site, or weakly/heterogeneously labeled, constraining model training, calibration, and external validation and amplifying sampling bias. Pipelines still depend on manual transcription or limited annotation, which further limits scale and reproducibility. Key metadata‐recording conditions, microphone placement, noise profiles, speaker demographics, comorbidities, and preprocessing decisions—are frequently under‐documented, and code release is inconsistent. Although some groups have begun releasing open resources and benchmarks, coverage across ages, languages, and clinical settings remains narrow; label schemas are not harmonized; and standardized reporting artifacts (e.g., dataset/model documentation) are not yet routine (Eni et al. 2025; Leland et al. 2023). These shortages and gaps impede cumulative science and slow clinical translation.

### 4.5 Ethics, Privacy, Fairness, and Governance

Child and in‐home audio involves vulnerable participants and highly intimate contexts, increasing ethical obligations around respect for persons, autonomy, privacy, and justice. Strict consent, data‐residency, and secondary‐use conditions determine who can participate and which evidence is admissible. Even strong anonymization can leave residual re‐identification risk in acoustic signals (Gadotti et al. 2024). As LLMs and self‐supervised models enter ASD speech workflows, model opacity and dataset imbalance can undermine accountability, contestability, and stakeholder trust (Keykha et al. 2024). Moreover, privacy‐preserving and local/edge processing create tensions with validity, reproducibility, and equitable coverage across languages, cultures, and sites, especially when data cannot be moved for cross‐site harmonization (Tajabadi et al. 2024). Unresolved governance issues include ownership and custodianship, lifespan of parental consent, cross‐border transfers, incidental findings, model updates over time, and third‐party processing.

### 4.6 Computational Efficiency and Deployment Constraints

State‐of‐the‐art deep learning, SSL, and transformer models are compute‐ and memory intensive. Long, noisy, multi‐speaker home recordings exacerbate latency and battery constraints for real‐time or on‐device use; edge‐constrained clinics face similar barriers (Chi et al. 2022; Lee et al. 2020). In privacy‐sensitive contexts, the need to process locally further burdens resource budgets, creating practical bottlenecks even when accuracy is adequate in research settings (Tajabadi et al. 2024).

**FIGURE 2:**
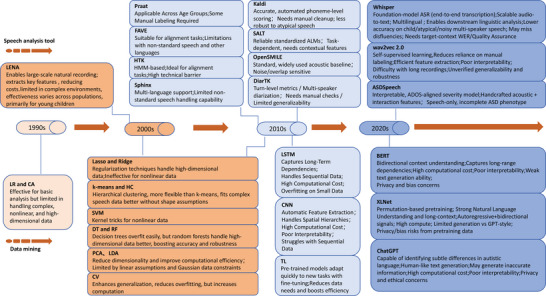
Historical development of automation technologies and data‐mining methods for ASD speech recognition (All abbreviations are defined at first mention in the text or in the Abbreviations and Glossary section). Abstract: This timeline summarizes the evolution of key computational tools and analytical methods deployed in autism speech research, charting the progression from early statistical models to contemporary self‐supervised and Transformer‐based artificial intelligence. Test: The figure is organized chronologically and by methodology. It begins with early tools like LENA and Praat alongside foundational methods (LR, CA). It then progresses through the 2000s‐2010s, showcasing the adoption of alignment toolkits (HTK, FAVE), robust ASR systems (Kaldi), and classical machine learning algorithms (SVM, RF, PCA). The modern era is characterized by deep learning sequence models (CNN, LSTM), self‐supervised speech encoders (Wav2vec 2.0), foundation‐model ASR (Whisper), and clinically‐oriented systems (ASDSpeech), culminating in the recent application of pretrained Transformer models and LLMs (BERT, XLNet, and ChatGPT) for semantic‐pragmatic analysis For each tool or method, key strengths and limitations are summarized to provide a balanced technological assessment.

Figure 2 summarizes the historical evolution of automation technologies and data‐mining methods for ASD speech recognition, highlighting the methodological shifts over time.

## 5 Review Limitations and Future Directions

### 5.1 Limitations

This review focused on methodological inflection points, rather than exhaustive retrieval or meta‐analysis. Heterogeneity in tasks and outcomes precluded quantitative pooling, and performance estimates across studies are not directly comparable. The evidence base remains concentrated in English, fluent‐speech cohorts with small, single‐site samples. Moreover, some evidence derives from preliminary or preprint reports. These constraints may limit transportability across languages, ages, and recording settings, and readers should interpret trends as indicative rather than definitive.

### 5.2 Future Directions: Three Strategies for Translation and Scale

We propose three complementary strategies that preserve our review's evidence base while specifying practical next steps.

#### 5.2.1 Optimization and Synergy of Existing Technologies

Rather than creating further standalone tools, a near‐term priority is to integrate mature toolchains and harmonize outputs. There are two practical dimensions to this strategy. First, complementary components such as LENA diarization, Praat/HTK/FAVE acoustic and alignment tools, and Kaldi‐ or wav2vec‐style ASR should be combined within coherent pipelines (Moffitt et al. 2022). Moreover, multimodal evidence should be leveraged for affect and pragmatics. Systematic reviews and multimodal training studies report that robust emotion recognition for ASD is enhanced by fusing speech with video and text streams, provided reporting standards are clear and there is cross‐task protocol alignment (Landowska et al. 2022; Olsson et al. 2025; Sangeetha et al. 2024). Second, heterogeneous outputs should be mapped onto a common metric panel for reporting and model evaluation: for example, accuracy or AUC for classification, WER/PER for ASR, and MAE or *R* ^2^ for continuous severity. Such harmonization would reduce brittle bespoke scripts, ease cross‐study comparison, and facilitate plugging new models into existing clinical workflows. Rigorous quality control—manual spot checks, standardized preprocessing, and transparent documentation of failure modes—remains essential (Kocak et al. 2025; Van Calster et al. 2024).

#### 5.2.2 Global Cooperation and Privacy‐Preserving Data Integration

Methodological advances will not generalize without larger, more diverse datasets. Cross‐country and cultural collaborations can address current gaps in language, age, and comorbidity by sharing protocol templates, task batteries (e.g., narration, conversation, repetition), and agreed‐upon dev/test splits (Hu et al. 2024; Lewerenz et al. 2024). Since unrestricted pooling of sensitive child speech data cannot be permitted, privacy‐preserving integration is crucial. Federated or distributed learning, secure enclaves, and synthetic‐data augmentation can allow models to benefit from multi‐site variability without centralizing raw audio (Tajabadi et al. 2024; Tanveer et al. 2025; Zhao et al. 2025). Moreover, clear data‐governance frameworks—covering consent, retention, secondary use, and data provenance transparency—are needed to ensure that global models remain accountable and equitable, especially for under‐resourced languages and communities (Tajabadi et al. 2024).

#### 5.2.3 Technological Innovation and Cross‐Domain Borrowing

We propose that this third strategy should include the following elements:

- Speech enhancement for real‐world audio: Advance deep multichannel speech enhancement and microphone‐array processing—beamforming, blind source separation, and learned spatial filters—to stabilize recognition in multi‐speaker, low‐signal‐to‐noise ratio (SNR) home recordings. Recent relevant work has explored dual‐microphone neural enhancement and array‐based methods (Wen et al. 2025; Tanveer et al. 2025; Natarajan et al. 2025).
- Label‐efficiency via hybrid annotation: Build efficient automatic and semi‐automatic annotation pipelines by combining supervised, semi‐supervised, and active learning, borrowing from high‐throughput biomedicine and label‐efficient medical AI frameworks (Jin et al. 2025; Zhao et al. 2025; Zhou and Yu 2025). Moreover, publish task taxonomies, error schemas, and inter‐rater reliability alongside codes and checkpoints to improve replicability (Zhou and Yu 2025; Zhang 2025).
- Address class imbalance or rare phenomena (e.g., echolalia variants): Combine classic perturbation‐based augmentation (e.g., altering rate, pitch, adding noise, or SpecAugment masking) with synthetic data generated via GANs (generative adversarial network) or TTS (text‐to‐speech). Such techniques do require strict controls and prospective cross‐dataset validation. Recent studies indicate that while generative augmentation can improve minority‐class performance, it is highly sensitive to distributional mismatch. Some studies even report degraded cross‐dataset detection when augmented data are not properly constrained (Aliouat and Djendi 2025; Ali et al. 2025; Woolsey et al. 2024).
- Explainability by design: Combine local/post‐hoc methods (e.g., LIME [Local Interpretable Model‐agnostic Explanations]; SHAP [Shapley Additive Explanations]) with clinically curated concepts (e.g., pause structure, pitch variability, turn‐taking) and, where feasible, embed domain knowledge into model architectures to stabilize reasoning (Jeon et al. 2024). Recent medical AI research advocates moving beyond purely post‐hoc explanations toward transparent, accountable, decision‐useful interpretability, with frameworks that jointly address transparency in development, built‐in interpretability, and carefully validated post‐hoc explanations (Mathew et al. 2025; Singh et al. 2025).
- Hardware acceleration—watching the horizon: Track, but do not yet deploy, emerging photonic AI accelerators (e.g., silicon‐photonic tensor cores and neuromorphic photonic chips for ultra‐fast, energy‐efficient deep learning) and quantum‐AI hardware developments as potential enablers of real‐time, low‐power pipelines. Concentrate on statistical rigor, reproducibility, and governance (Fayza et al. 2025; Ning et al. 2025).
- Edge‐ready, lightweight inference: Pursue compact models and distributed/edge computing for low‐latency, on‐device processing in clinics and homes, leveraging tiny machine learning (TinyML) and federated edge‐learning frameworks that respect resource constraints and privacy in healthcare settings (Heydari et al. 2025; Mahmood et al. 2025; Tajabadi et al. 2024).

Beyond screening and diagnosis, automated speech and language measures may be especially well‐suited for longitudinal monitoring of development and response to intervention. Compared with between‐subject diagnostic classification, within‐person tracking can leverage an individual baseline and may be less sensitive to heterogeneity in language level, context, and comorbidities. Frequent, low‐burden samples (e.g., brief telehealth interactions or periodic naturalistic recordings) could quantify change in vocalization rate, conversational turns, lexical diversity, prosody, and interactional timing, supporting measurement‐based care and rapid feedback in clinical and educational settings.

Collectively, these recommendations retain the strengths of current practice while prioritizing transportability, clinically anchored endpoints, standardized evaluation, and responsible deployment. When paired with external, cross‐site validation and public code/model documentation, they provide a concrete path from proof‐of‐concept to routine clinical use. These approaches may be especially impactful for scalable, low‐burden monitoring of progress over time, complementing (rather than replacing) gold‐standard diagnostic evaluations.

## Author Contributions

**Rongjie Mao**: conceptualization, investigation, writing – original draft. **Yuncheng Zhu**: supervision (guiding the review framework), writing – review and editing.

## Funding

The work was supported by the Health System Key Discipline of Shanghai (2024ZDXK0013), the Key Support Program for Clinical Specialty of Hongkou District Health Commission (HKLCFC202408), the Scientific Research Project of Hongkou District Health Commission (2301‐05,2401‐06), Granted by the Clinical Research Center for Mental Health, School of Medicine, Shanghai University (SHU‐HMH‐202508), Scientific Research Project of Hongkou District Health Commission (HKZYY‐2025‐05).

## Supporting information



Table S1. Automated speech analysis tools used in ASD speech research: components, contexts, evidence, and recommended use.

Table S2. Data mining and machine learning methods for ASD speech analytics: representative studies, strengths, limitations, and integration scenarios.

## Data Availability

The authors have nothing to report.
