# Supplementary material for: Use of Automation Technologies and Data Mining in Speech Recognition for Autism
Source: Brain Behav. 2026 Jan 28;16(2):e71229. doi: 10.1002/brb3.71229 (PMC12848528; doi:10.1002/brb3.71229)
Supplement: Supplementary file 1 — Table S1. Automated speech analysis tools used in ASD speech research: components, contexts, evidence, and recommended use. [file BRB3-16-e71229-s001.docx]

**Table S1. Automated speech analysis tools used in ASD speech research: components, contexts, evidence, and recommended use**

| **Tool / Component type** | **Speech context and typical features extracted** | **Representative studies(first auththor,year)** | **Population and age (ASD)** | **Main findings (selected metrics)** | **Strengths** | **Limitations** | **Best‑fit use‑case / Integration** |
| --- | --- | --- | --- | --- | --- | --- | --- |
| LENA | Naturalistic speech; interactional counts (AWC, CVC, CTC) and derived acoustics (rhythm, spectral tilt, pitch control) | Oller 2010; Yoder 2013; Woynaroski 2017; Jones 2019; McDaniel 2020; Putnam 2025 | Toddlers/young children (≈16-48 months); performance drops at ≥5 years; sibling cohorts used | Sensitivity 0.75 / Specificity 0.98; ICC = 0.91; IVD/ACPU‑C+V predict later vocabulary; accuracy <50% in children with ASD aged ≥5 years; human‑coded measures are stronger predictors | Ecologically valid, scalable, feasible for longitudinal monitoring | Susceptible to noise/overlapping speakers; poorer accuracy in older children | Early monitoring; pair with Praat or Sphinx for detail/segmentation |
| Praat | ADOS interviews, narratives; F0, rate, rhythm, intensity, jitter, shimmer, HNR, CPP, formants | Bone 2014; Patel 2020; Wynn 2022 | Children and adults | Prosodic/voice features correlate with symptom severity; rate is strongest ASD predictor; lower articulatory precision | Transparent, fine‑grained, widely adopted | Manual quality control; sensitive to noise/overlap | High‑resolution acoustic phenotyping; integrate with HTK/FAVE or DNNs |
| HTK | Social interaction / ADOS speech; time‑locked segmentation for downstream acoustics | Bone 2014 | Children(5.8-14.7y) | Alignment enabled extraction of features (e.g., pitch slope) correlated with ASD severity | Temporal precision; standard in phonetic pipelines | Reduced accuracy for child/atypical voices | Alignment backbone before Praat/eGeMAPS |
| FAVE | Narrative retelling task / conversation; vowel space (F1/F2), prosody and rhythm | Patel 2020 | Children to adults | Slower rate and larger final F0 excursion in ASD; speech rate is best single predictor | Efficient vowel/formant analysis | Reduced reliability with non‑English data | English vowel/tempo analyses; combine with Praat |
| CMU Sphinx | Naturalistic speech (often paired with LENA); ASR transcripts (pre-segmented input); MFCC-based acoustic model | Woynaroski 2017; McDaniel 2020; Markfeld 2023 | Toddlers; early‑risk siblings | IVD/ACPU‑C+V stable and predictive; AVA‑DA weaker; stability varies across groups | Scales to long recordings; open‑source | Accuracy drops in noisy/multi‑speaker environments | Large‑scale automated counts/transcripts; integrate with LENA or NLP models |
| Kaldi ASR | Nonword or sentence repetition task; frame alignments, PER, WER | Asgari 2017; Gale 2019 | Children 5-8/6-9 y | MAE = 0.04; *r* = 0.85 with clinician ratings; PER ≈ 19.8%; transfer learning reduced WER 29.4%→26.2% | Highly adaptable; supports transfer learning | Needs manual removal of irrelevant segments | Automated scoring/Quality Assurance of NWR; pair with regression/Machine Learning |
| AutoSALT | ADOS interview, conversation, narration; MLUM, NDWR, CPM, unintelligible/repetition proportions | MacFarlane 2023 | 6-23 y; ASD cohorts | CCC ≈ 0.73-0.88; significant differences in ALM by task (*p* < .001) | Standardized ALMs; facilitates comparisons | Task/context performance variability; manual transcripts often needed | Language‑sample pipelines; combine with acoustic features |
| OpenSMILE + eGeMAPS | Nonword or sentence repetition task; infant recordings; prosody and voice quality (MFCC, jitter, shimmer, HNR, energy) | Lee 2020; Beccaria 2022 | Children; infants | Bi‑LSTM accuracy ≈ 68% in infants; SVM 83% (recall 100%, precision 67%); decision tree 100% recall (96% precision) | Widely used, comparable baselines | Sensitive to noise; limited generalization | Baseline paralinguistic extraction; feed SVM/DNN |
| DiarTK | ADOS‑2 interviews; turn-taking, initiation, durations; MFCC features | Sadiq 2019 | Small clinical cohorts (ASD) | CNN/LSTM predicted ADOS‑2 Social Affect (*R*² = 0.402) | Enables multi‑speaker analysis | Manual annotation burdens; small samples | Preprocessing for ADOS/social interaction corpora |
| wav2vec 2.0 | Social interaction; semi‑structured child speech; learned phonetic embeddings | Chi 2022; Eni 2025 | Children 3-12 y | Accuracy 76.9%; robust estimation of ADOS‑2 Social Affect | Label‑efficient; strong transfer; reduces manual features | Needs adaptation for noise/atypical voices | End‑to‑end pipelines; combine with TRILLsson or text features |
| TRILLsson + cascaded multimodal frameworks | Severity assessment from raw audio without transcripts; suprasegmental embeddings + ASR text | Mun 2025 | Pediatric ASD | Best agreement with clinician severity (ρ ≈ 0.56); improved atypical phonation recognition | Captures prosody; transcript‑free | Needs external validation; compute‑intensive | Fusion with wav2vec 2.0 + KLUE/RoBERTa |
| Whisper ASR (OpenAI) | Classroom child-teacher interaction; ASR transcripts for communication profiling (pragmatics/syntax/semantics; echolalia) | Muna 2025 | Children with mild and moderate verbal ASD; teachers (age not reported) | WER: mild 0.56-10% (median≈5%); moderate 13-46% (median≈23%); GPT-4o diarization accuracy 93.9% (mild) vs 89.2% (moderate); total analysis time −89.1% | Off-the-shelf ASR; efficient transcription backend for diarization-enabled pipelines | WER rises and varies with severity; requires in-domain error profiling | Use for fast transcription with diarization; report WER and account for ASR uncertainty downstream |
| Text Transformers / LLMs(e.g., BERT, ChatGPT) | Narrative retelling task; semantic‑pragmatic cues (grammar, syntax, semantics, narrative coherence, echolalia/pronoun use) | Wawer 2022; Themistocleous 2024;Hu 2024 | Children 4-10 y; adolescents; adults | BERT enhanced context-based semantic depth, achieving around 96%  accuracy; ChatGPT > BERT/XLNet/ALBERT (+12-22 percentage points, F1) | Captures semantics and pragmatics; strong fusion gains | Dependent on transcript quality; privacy/interpretability concerns; high compute and memory footprint | Semantic‑pragmatic profiling; fuse with acoustic/SSL features |

Abbreviations not defined in the main text are explained below; all others are defined at first mention in the article.

ICC:intraclass correlation coefficient;AVA-DA:Automated Vocalization Analysis-Developmental Age;AWC:adult word count;CPP:cepstral peak prominence;CPM: communication units per minute;CTC: conversational turn count;CVC: child vocalization count;KLUE:Korean Language Understanding Evaluation；NLP: natural language processing;NWR: non-word repetition;RoBERTa: A Robustly Optimized BERT Pretraining Approach.
