# Supplementary material for: Use of Automation Technologies and Data Mining in Speech Recognition for Autism
Source: Brain Behav. 2026 Jan 28;16(2):e71229. doi: 10.1002/brb3.71229 (PMC12848528; doi:10.1002/brb3.71229)
Supplement: Supplementary file 2 — Table S2. Data mining and machine learning methods for ASD speech analytics: representative studies, strengths, limitations, and integration scenarios. [file BRB3-16-e71229-s002.docx]

**Table S2. Data mining and machine learning methods for ASD speech analytics: representative studies, strengths, limitations, and integration scenarios**

| **Data‑mining / machine-learning method** | **Speech context and extracted features** | **Representative studies（first auththor,year）** | **Population / Age (ASD)** | **Main findings and key metrics** | **Strengths** | **Limitations** | **Best‑fit use‑case / Integration** |
| --- | --- | --- | --- | --- | --- | --- | --- |
| LR; CA | Early manual pipelines using clinician‑coded language/speech measures; task‑based behavioral and linguistic features | Eaves 1994; Eisenmajer 1996 | Mixed pediatric cohorts; small samples | Enabled early quantitative patterning of ASD subtypes and features; efficient/interpretable but limited for complex, nonlinear speech patterns | Simple, interpretable; low compute; baseline for Knowledge Discovery in Databases in ASD | Relies on handcrafted features and small manual datasets; weak for nonlinear/temporal dynamics | Historical baselines; feature screening before modern machine learning; combine with PCA or LDA to reduce dimensionality |
| PCA/ LDA + MLR | Naturalistic speech; developmental acoustics (rhythm, syllabicity, spectral tilt, pitch‑control, duration) | Oller 2010 | Toddlers 16–48 months | ASD vs. TD: Sensitivity 0.75; Specificity 0.98 (LOOCV) using LENA‑derived features | Leverages interpretable low‑dimensional structure; works with limited labels | Age‑restricted generalizability; sensitive to recording noise/overlap | Early screening with LENA features; pair with Praat/eGeMAPS for richer acoustics |
| Regularized regression (ridge, lasso) and linear SVR | Nonword or sentence repetition task; phoneme errors and accuracy; ASR‑aligned frames | Asgari 2017 | Children 5–8 y (ASD/SLI/TD) | Automated NWR scoring: MAE = 0.04; *r* = 0.85 vs. clinician ratings; PER ≈ 19.76% | Controls overfitting; continuous scoring; small‑sample friendly | Needs manual removal of irrelevant segments; limited by ASR errors | Objective scoring of non-word/sentence repetition; feed scores into downstream prognostic models |
| SVMs for pragmatic/text features | Narrative retelling task; word alignment, log‑odds lexical atypicality; pragmatic indices | Prud'hommeaux 2012 | Children 4–8 y (ASD/TD/SLI) | Non‑ASD vs. ASD: AUC ≈ 0.72 (combined features) | Handles nonlinear boundaries; robust on small to medium samples | Requires high‑quality transcripts; limited semantic depth | Text branch in multimodal pipelines; combine with BERT embeddings for semantics |
| Cross‑linguistic SVM on rhythm vs. intonation | Narrative retelling task; rhythm (envelope spectrum, IMFs) vs. intonation (F0 contours) | Lau 2022 | Children/young adults (English and Cantonese groups) | Rhythm AUC = 0.900 (English), 0.962 (Cantonese); Intonation AUC = 0.695/0.620 | Highlights stable cross‑linguistic markers (rhythm) | Wide age range; gender imbalance; language effects | Cross‑site generalization; choose rhythm features for multilingual screening |
| RF / DT ensembles | Social interaction (SIT); acoustics (F0, HNR, MFCC, jitter/shimmer, energy) + facial features | Drimalla 2020 | Adults 22–62 y | Accuracy 73%; Sensitivity 67%; Specificity 79% | Nonparametric; good with mixed feature types; feature importance available | Moderate accuracy; site/age variability; potential class imbalance | Multimodal screening; combine with calibration and external validation |
| HMM/ Temporal modeling | ADOS-2 interview; forced alignment and segmental timing; prosody trajectories (e.g., turn‑end pitch slope) | Bone 2014; Prud'hommeaux 2012 | Children 4-14.7y | Temporal alignment enabled extraction of severity‑linked cues (e.g., flatter pitch slope, r ≈ −0.68) | Captures sequence structure; precise alignment for feature timing | Adult‑trained models degrade on child/atypical voices; manual quality control needed | Alignment backbone before acoustic feature extraction; adapt models for child speech |
| k‑means clustering + multivariate pipeline | Nonword or sentence repetition tasks; acoustics: f0, formants (F1–F4), HNR, jitter, shimmer | Briend 2023 | Children 6.3–12 y | ASD vs. TD: accuracy 91%; sensitivity 89%; specificity 94% (Monte‑Carlo CV) | Simple/efficient unsupervised structure discovery; robustness via resampling | Small, narrow cohort; needs broader validation | Triage/class discovery; integrate as pre‑step before supervised models |
| Time‑varying harmonic model + SVM | ADOS-2 interview; prosodic/voice quality set (pitch, jitter, shimmer, HNR, loudness, spectral entropy) | Asgari 2021 | Children 7.8–15.3 y | All features: AUC = 82.23%; Friends/marriage task: AUC = 83.04% | Physiologically motivated; good discriminability | Small male‑skewed controls; task/context sensitivity | Prosody‑centric pipelines; task‑specific screening |
| Multimodal fusion (ALM + AVM) with SVM/DNN | ADOS interviews; ALM (MLUM, NDWR, etc.) + AVM (acoustic prosody/quality) | MacFarlane 2022 | Children 7–17 y (English, typical IQ) | Combined AUC 0.9205; accuracy 86.71%; sensitivity 89.77%; specificity 82.86% (LOOCV) | Fusion > unimodal; clinically meaningful endpoints | Cohort narrow; language/IQ bias; small sample | Best for clinic‑style speech; extend to multilingual with adapted features |
| CNN / LSTM (deep sequence models) | ADOS‑2 interviews; diarized speech; MFCC and SED features | Sadiq 2019 | Clinical cohorts (size limited) | Predicted ADOS‑2 Social Affect: *R*² = 0.402 | Learns temporal dynamics; higher automation | Data‑hungry; manual labels; overfitting risk in small cohorts | Sequence modeling of interactional speech; pair with augmentation and cross‑site CV |
| Autoencoder + Bi‑LSTM | Infant recordings; openSMILE/eGeMAPS (88 acoustic features): F0, formants, MFCCs, energy | Lee 2020 | Infants 6–24 months (ASD/TD) | Bi‑LSTM accuracy ≈ 68.18%; sensitivity ≈ 68.69% | Unsupervised precompression + temporal modeling | Imbalanced, noisy data; limited interpretability | Early‑risk screening; use with careful quality control and balanced sampling |
| TL for ASR (Kaldi DNN/GMM‑HMM) | Nonword or sentence repetition task; MFCC, i‑vectors; frame‑level alignments; WER/PER | Gale 2019 | Children 6–9 y (ALI/ALN/SLI) | WER 29.38%→26.21% with augmentation; diagnosis‑specific WER: ALN 20.51%, ALI 29.95%, SLI 36.60% | Leverages adult speech pretraining; gains with augmentation | Age/domain shift harms generalization; best with narrow age bands | Automated scoring under limited data; pair with transfer/augmentation |
| Gradient boosting / XGBoost with Transformer embeddings | Nonword or sentence repetition task; grammar, syntax, semantics, text complexity; contextual embeddings (BERT) | Themistocleous 2024 | Children 4–10 y | Distinguishing ASD from TD: accuracy ≈ 96% (5‑fold CV) | Captures long‑range semantics; strong results in pediatric narratives | Transcript quality dependence; wide age range; external validity pending | Semantic‑pragmatic profiling; combine with acoustic features for fusion gains |
| Deep text embeddings (ELMo/USE) + SVM / XGBoost | Narrative retelling task; semantic abstraction, sentiment, coherence | Wawer 2022 | 7.1–24.6 y (balanced ASD/TD) | Outperformed human raters (sensitivity/specificity 0.72/0.68 vs. 0.56–0.60/0.60–0.64), but below ADOS‑2/SCQ(Social Communication Questionnaire) | Automated high‑level text features; good small‑sample behavior | Below clinical gold standards; explainability limits | Text branch for multimodal models alongside acoustics |
| Self‑supervised speech encoders (wav2vec 2.0) | Social interaction / semi‑structured child speech; raw‑audio embeddings capturing phonetics | Chi 2022 | Children 3–12 y | Accuracy 76.9% for wav2vec 2.0, vs. 69.7% for RF and 79.3% for CNN | Label‑efficient; robust phonetic representation | Needs adaptation for noisy/atypical child speech; interpretability | End‑to‑end pipelines; combine with TRILLsson / text encoders; ASD‑specific fine‑tuning |
| LLMs (ChatGPT) vs. BERT/XLNet/ALBERT | Social interaction; echolalia, stereotyped media quoting, pronoun shifts, social phrasing | Hu 2024 | Adults ((Not specified)) | ChatGPT accuracy 81.82% vs. BERT 63.92%; F1 79.89% vs. 61.87% (precision/recall also higher) | Strong pragmatic detection; zero/low‑shot capability | Privacy/ethics; transcript quality; small samples; opacity; high compute and memory footprint; compute/latency constraints for clinical edge deployment | Clinical‑style narrative analysis; pair with privacy‑preserving and XAI methods |

Abbreviations not defined in the main text are explained below; all others are defined at first mention in the article.

CA:cluster analysis;CV: cross-validation;IMFs: intrinsic mode functions；i-vectors: low-dimensional utterance-level representations derived from factor analysis, widely used in speaker recognition;MLR:multiple linear regression;RF:random forest;SCQ: Social Communication Questionnaire;SED: speech activity and segmentation features;SIT: Simulated Interaction Task;SLI/ALI/ALN: Specific Language Impairment / Autism + Language Impairment / Autism without Language Impairment;SVR: support vector regression;XAI: explainable artificial intelligence
